# Editorial: Drugging p53 for non-cancer diseases

**DOI:** 10.3389/fphar.2022.1101742

**Published:** 2022-11-30

**Authors:** Fan Jiang, Chun-Guang Li, Sai-Wang Seto

**Affiliations:** ^1^ Shandong Key Laboratory of Cardiovascular Proteomics and Department of Geriatric Medicine, Qilu Hospital, Cheeloo College of Medicine, Shandong University, Jinan, China; ^2^ NICM Health Research Institute, Western Sydney University, Westmead, NSW, Australia; ^3^ Department of Applied Biology and Chemical Technology, The Hong Kong Polytechnic University, Hong Kong SAR, China; ^4^ Research Centre for Chinese Medicine Innovation, The Hong Kong Polytechnic University, Hong Kong SAR, China

**Keywords:** p53, non-cancer disease, small molecule, activator, inhibitor, ligand, drug discovery, drug target

It has been once believed that p53 is ‘undruggable’ by small-molecule ligands because it is a transcription factor, which generally lacks conventional ligand binding pockets (Henley and Koehler, 2021). Nonetheless, recent advancements in our knowledge about the molecular regulation of p53 protein have enabled the discovery of means to target this critical transcription factor by small molecules, rendering the p53 pathway to be practically “druggable” (Sanz et al., 2019; Hassin and Oren, 2022). Most of these strategies are indirect in fact, *i.e.* the ligands do not take effects by binding to p53 *per se* but *via* interactions with endogenous modulators of p53. These compounds include Mdm2 inhibitors, Mdmx inhibitors, Sirt1/2 inhibitors, inhibitors of the deubiquitinase USP7, and inducers of the nucleolar stress response (Sanz et al., 2019; Cui et al., 2021).

Undoubtedly, novel p53-drugging strategies will benefit the clinical treatment of cancers (Sanz et al., 2019). On the other hand, p53 also plays important roles in modulating the pathogenesis of an array of non-cancer diseases, including cardiovascular disease, neural degenerative disorders, inflammatory and autoimmune diseases. Being a key transcription factor ubiquitously expressed in the body, p53 has been shown to impact the expression of over 60 genes involved in various aspects of cell biology (apoptosis, senescence, cell cycle regulation, and maintenance of genome stability). Therefore, p53 is thought to influence the physiology and/or pathophysiology virtually in all organs. Supporting this notion, experimental and clinical evidence suggests that p53 activators may also have therapeutic potentials related to non-canonical p53-mediated responses, such as regulation of immunity/inflammation (Hassin and Oren, 2022) and repression of proliferative vascular diseases (Cui et al., 2021).

In this Research Topic, Wang et al. reported their work on identifying the molecular mechanisms underlying the anti-inflammatory effects of CX-5461, a novel selective RNA polymerase I inhibitor which induces nucleolar stress and p53 activation (phosphorylation), in macrophages. This study extended their previous findings that CX-5461, in addition to its anti-tumor activity, exhibited significant anti-inflammatory and immunosuppressive effects (Dai et al., 2018; Xu et al., 2021; Pan et al., 2022). The authors utilized contemporary systemic biology techniques (genome-wide RNA sequencing). They demonstrated that CX-5461 principally induced a molecular signature related to cell cycle inhibition in lipopolysaccharide- and interferon-γ-primed macrophages, evidenced by downregulation of a cluster of genes encoding cell cycle mediators and concomitant upregulation of those encoding cell cycle inhibitors. However, CX-5461 did not induce a systemic anti-inflammatory transcriptional program in macrophages, although some pro-inflammatory genes, such as interleukin-1β and gp91phox NADPH oxidase, were downregulated by CX-5461. Consistent with their previous observations (Dai et al., 2018; Xu et al., 2021; Pan et al., 2022), this study confirmed a central role of p53 in orchestrating the molecular responses in macrophages to CX-5461 treatment. The authors suggested that limiting cell proliferation was the predominant mechanism of the inhibitory effects of CX-5461 on macrophage-mediated inflammation, and these data might provide a molecular framework for understanding the mechanisms underlying the anti-inflammatory properties of CX-5461.

In contrast to the undisputed tumor suppressor role in cancer cells, p53 appears to be a double-edged sword in non-cancer diseases. Chan et al. provided a review article in which they summarized the divergent effects of p53 in endothelial and vascular smooth muscle cells. Further, they discussed the specific roles of p53 in various cardiovascular disorders. Evidence supports that exaggerated activation of p53 may contribute to endothelial dysfunctions. However, at least several gene knockout studies suggest that endogenous p53 plays a protective role during atherogenesis *in vivo*. Moreover, p53 activation exhibits beneficial effects in the pathogenesis of pulmonary arterial hypertension, highlighting the notion that drugging the p53 pathway may represent a novel strategy to prevent the development of proliferative vascular remodeling (Cui et al., 2021).

The study by Gao et al. aimed to discover novel SIRT1 (Sirtuin 1) activators. Using structure and ligand-based virtual screening, they identified a lead compound (named M1) exhibiting a potent SIRT1 activation effect. Based on M1, they further synthesized a series of novel naphthofuran derivatives with SIRT1 activating properties. Of note, these compounds displayed some p53 inhibiting effects (reduction in p53 acetylation with no change in total p53 protein level) in high glucose-challenged HK-2 cells (a human kidney proximal tubular epithelial cell line). In another study, Huang et al. reported that in human conjunctival fibroblasts, treatment with the selective ATR (ataxia telangiectasia and Rad3-related) inhibitor AZD6738 (aka ceralasertib) upregulated the mRNA level of p53. This effect was accompanied by inhibition of cell proliferation and reduction in the expression of collagens. These findings are consistent with our recent results showing that activation of the p53 pathway by CX-5461 has anti-fibrotic effects in cardiac fibroblasts (Pang et al., 2021). Unfortunately, Huang et al. did not examine the effects of AZD6738 on either p53 protein levels or p53 phosphorylation. Also, it should be noted that the anti-fibrotic effects of AZD6738 appeared to involve other signaling pathways apart from p53. Based on their findings, the authors argued that AZD6738 may become a potential therapeutic option to prevent subconjunctival scarring caused by trabeculectomy surgery.

Challenges remain in the development of clinically usable p53-targeting drugs. In addition to the problem of frequent p53 gene mutation in cancers, occurrence of unwanted side effects (such as hematopoietic repression) is a major concern for patients with non-cancer diseases. Also, the target selectivity of the currently available compounds in this class is often controversial (Cui et al., 2021). Therefore, more pre-clinical studies are still required to identify promising lead compounds for drugging the p53 pathway.

## References

[B1] Cui X. Pan G. Chen Y. Guo X. Liu T. Zhang J. (2021). The p53 pathway in vasculature revisited: A therapeutic target for pathological vascular remodeling? Pharmacol. Res. 169, 105683. 10.1016/j.phrs.2021.105683 34019981

[B2] Dai C. Sun M. Wang F. Zhu J. Wei Y. Guo X. (2018). The selective RNA polymerase I inhibitor CX-5461 mitigates neointimal remodeling in a modified model of rat aortic transplantation. Transplantation 102, 1674–1683. 10.1097/TP.0000000000002372 30247451

[B3] Hassin O. Oren M. (2022). Drugging p53 in cancer: one protein, many targets. Nat. Rev. Drug Discov., 1–18. 10.1038/s41573-022-00571-8 PMC9549847 36216888

[B4] Henley M. J. Koehler A. N. (2021). Advances in targeting ‘undruggable' transcription factors with small molecules. Nat. Rev. Drug Discov. 20, 669–688. 10.1038/s41573-021-00199-0 34006959

[B5] Pan G. Zhang J. Han Y. Chen Y. Guo X. Cui X. (2022). CX-5461 is a potent immunosuppressant which inhibits T cell-mediated alloimmunity via p53-DUSP5. Pharmacol. Res. 177, 106120. 10.1016/j.phrs.2022.106120 35131482

[B6] Pang S. Chen Y. Dai C. Liu T. Zhang W. Wang J. (2021). Anti-fibrotic effects of p53 activation induced by RNA polymerase I inhibitor in primary cardiac fibroblasts. Eur. J. Pharmacol. 907, 174303. 10.1016/j.ejphar.2021.174303 34217709

[B7] Sanz G. Singh M. Peuget S. Selivanova G. (2019). Inhibition of p53 inhibitors: Progress, challenges and perspectives. J. Mol. Cell Biol. 11, 586–599. 10.1093/jmcb/mjz075 31310659 PMC6735775

[B8] Xu X. Feng H. Dai C. Lu W. Zhang J. Guo X. (2021). Therapeutic efficacy of the novel selective RNA polymerase I inhibitor CX-5461 on pulmonary arterial hypertension and associated vascular remodelling. Br. J. Pharmacol. 178, 1605–1619. 10.1111/bph.15385 33486761 PMC9328314

